# L-Cysteine Upregulates Testosterone Biosynthesis and Blood–Testis Barrier Genes in Cultured Human Leydig Cells and THP-1 Monocytes and Increases Testosterone Secretion in Human Leydig Cells

**DOI:** 10.3390/biom14091171

**Published:** 2024-09-18

**Authors:** Jeffrey Justin Margret, Sushil K. Jain

**Affiliations:** Department of Pediatrics, Louisiana State University Health Sciences Center, Shreveport, LA 71103, USA; jeffrey.justinmargret@lsuhs.edu

**Keywords:** blood–testis barrier, Claudin, *CYP11A1*, L-cysteine, Leydig cells, male infertility, testosterone, THP-1 monocytes

## Abstract

Leydig cells are the primary source of testosterone or androgen production in male mammals. The blood–testis barrier (BTB) maintains structural integrity and safeguards germ cells from harmful substances by blocking their entry into the seminiferous tubules. L-cysteine is essential to the production of glutathione, a powerful antioxidant crucial to protecting against oxidative stress-induced damage. Animal studies have demonstrated the protective effect of L-cysteine in preventing testicular damage caused by chemicals or radiation. This study examines whether L-cysteine enhances the expression of testosterone biosynthesis and the BTB genes in human Leydig cells and THP-1 monocytes. The Leydig cells and THP-1 monocytes were treated with L-cysteine for 24 h. RNA was extracted following treatment, and the gene expression was analyzed using quantitative RT-PCR. Testosterone levels in the cell supernatant were measured using an ELISA kit. L-cysteine treatment in Leydig cells significantly upregulated the expression of *CYP11A1* (*p* = 0.03) and the BTB genes *CLDN1* (*p* = 0.03), *CLDN11* (*p* = 0.02), and *TJP1* (*p* = 0.02). Similarly, L-cysteine significantly upregulated the expression of *CYP11A1* (*p* = 0.03) and *CYP19A1* (*p* < 0.01), and the BTB genes *CLDN1* (*p* = 0.04), *CLDN2* (*p* < 0.01), *CLDN4* (*p* < 0.01), *CLDN11* (*p* < 0.01), and *TJP1* (*p* = 0.03) in THP-1 monocytes. Further, L-cysteine supplementation increased the testosterone secretion levels in human Leydig cells. The findings suggest that L-cysteine supplementation could be used as an adjuvant therapy to promote the integrity of the BTB genes, testosterone biosynthesis and secretion, and the maintenance of testicular functions, which in turn mitigates the risk of male infertility.

## 1. Introduction

Leydig cells are found in the interstitial compartment of the testis and serve as the main site for the biosynthesis and secretion of testosterone or androgens in male mammals [1,2]. Their importance lies in their role in sperm production, the regulation of sexual development, and the maintenance of secondary sexual characteristics and behaviors. Testosterone levels are significantly elevated in the testes compared to the bloodstream [3]. A reduction in intratesticular testosterone levels has been linked to impaired spermatogenesis, which is correlated with diminished expression of crucial proteins responsible for germ cell regulation [4]. Leydig cells express genes related to steroidogenesis [5]. The cholesterol side chain cleavage enzyme (CYP11A1) is a key enzyme in the testosterone synthesis pathway. In Leydig cells, cholesterol is hydroxylated by CYP11A1, which undergoes a series of reactions that eventually produce testosterone [6]. Decreased expression of essential proteins, such as CYP11A1, has been linked to decreased intratesticular testosterone levels and associated defects in spermatogenesis [4]. Defects in the *CYP11A1* gene cause pseudohermaphroditism and lipoid congenital adrenal hyperplasia [7,8]. Serum testosterone levels decline progressively with aging in rodents and humans [9].

The spermatogenic cells are interconnected and this dynamic relationship is regulated by tight junctions (TJs) and gap junctions (GJs). The TJ is important for the formation and function of the blood–testis barrier (BTB) [10]. During the active phase of spermatogenesis, germ cells differentiate and travel across the BTB. This process is unequivocally dynamic, with cells projecting to contact others, and adhesion molecules being rearranged precisely to admit the passage of germ cells without affecting barrier permeability [11]. Claudins (CLDN1, CLDN2, CLDN4, CLDN11, and CLDN15), zonula occludens-1 (ZO-1 or TJP1), and occludin (OCLN), a group of cell junctional proteins, serve as the backbone of the TJ. Claudin family members perform dual roles; some have barrier activities, while others mediate the permeability of small molecules and ions [12]. Defects in these proteins can cause BTB dysfunction, which may elicit an immune response against meiotic and postmeiotic cells, leading ultimately to spermatogenic failure and male infertility. Increased apoptosis and irregular tight junction (TJ) development are linked with claudin-11, occludin, and ZO-1 in cases of nonobstructive azoospermia [13]. In addition, the function of the BTB may also be compromised due to the defects in the genes that regulate the formation and function of cell junctions [14].

The amino acid L-cysteine is a critical building block required for the synthesis of proteins. L-cysteine acts as a precursor for glutathione biosynthesis, an important antioxidant. The reduced form of glutathione (GSH) plays a fundamental role in the organism’s defense against damage caused by oxidative stress. GSH is abundantly present in Leydig cells, and it safeguards cellular macromolecules from reactive oxygen and nitrogen species while directly neutralizing free radicals. With the aging of Leydig cells, there is a decline in GSH levels, resulting in heightened oxidative stress and diminished testosterone synthesis [15]. In animal studies, the antioxidant effect of N-acetyl-L-cysteine (NAC) reduces oxidative stress and protects against chromium-induced oxidative damage in mouse testis [16], treatment with sodium fluoride in rat testis [17], and reduces the damage caused by various chemicals and radiation to testicular cells [18,19,20,21]. In humans, oral supplementation with NAC improves sperm parameters and reduces oxidative stress in infertile males [22].

Oxidative stress is one of the primary factors that cause damage to Leydig cells by initiating lipid peroxidation and apoptosis, impairing mitochondrial function, and decreasing testosterone synthesis [23]. Dysregulation in Leydig cell function and BTB integrity ultimately leads to male infertility. Elevating gene expression could improve testosterone production, strengthen the integrity of the BTB, and alleviate the impacts of male infertility. No previous study has examined whether the protective effects of L-cysteine are mediated at the level of upregulation of BTB genes or/and testosterone biosynthesis genes and secretion. This study has examined the hypothesis that the expression of testosterone-regulatory and BTB genes and testosterone secretion are upregulated by L-cysteine. We treated both the human Leydig cells and THP-1 monocytes with L-cysteine to examine the expression of testosterone regulatory genes (*CYP11A1* and *CYP19A1*) as well as BTB genes (*CLDN1*, *CLDN2*, *CLDN4*, *CLDN11*, *CLDN15*, *TJP1*, and *OCLN*). 

## 2. Materials and Methods

### 2.1. Cell Culture Treatment

The human THP-1 monocyte cell line was obtained from the American Type Culture Collection (ATCC, Manassas, VA, USA) and maintained as described previously [24,25]. Cells (1 × 10^6^/mL) were pretreated with different concentrations of L-cysteine (500–1000 µM) for 24 h. Control cells were maintained with a normal glucose concentration (7 mM). Viability of cells was determined using the Alamar Blue reduction bioassay (Alamar Biosciences, Sacramento, CA, USA). 

The human Leydig cells (HLCs) were obtained from ScienCell Research Laboratories (Carlsbad, CA, USA). They were cultured in poly-L-lysine (PLL)-coated flasks with Leydig cell medium (ScienCell) and maintained according to the manufacturer’s protocol. Leydig cells have a limited expansion capacity, which makes them unsuitable for long-term culture, so they were treated with 500 µM L-cysteine for 24 h. Both the cell lines were used as per the institutional biosafety guidelines (#B 95-230). 

### 2.2. Testosterone Assay

After treatment, the cell supernatant was collected in an Eppendorf tube and preserved at −80 °C. The testosterone levels in the human Leydig cells and THP-1 monocytes were assessed using a commercially available ELISA kit (R&D Systems, MN, USA). First, 100 µL of cell culture supernatant was used for the assay. All necessary controls and standards as outlined by the manufacturer’s instructions were implemented. The absorbance was read at 450 nm. Each sample was analyzed in duplicate simultaneously, and the concentration was determined using a standard curve along with suitable blanks. 

### 2.3. RNA Extraction and qPCR

Following treatment, the cells were harvested, and total RNA was extracted using an RNeasy Mini Kit (Qiagen, Hilden, Germany). The human Leydig cells were trypsinized and the lysate was transferred to the column for RNA isolation. The concentration and quality of the isolated RNA were measured on a NanoDrop spectrophotometer (Thermo Scientific, Waltham, MA, USA). cDNA was synthesized from 1 µg RNA using a High-Capacity RNA-to-cDNA Kit (Applied Biosystems, Waltham, MA, USA) in a 20 µL reaction volume and then incubated at 37 °C for 60 min followed by 95 °C for 5 min. Quantitative real-time PCR was performed to determine the expression of mRNA using RT^2^ SYBR Green (Qiagen) in an Opus 384-2 Real-Time System (BioRad, Hercules, CA, USA). Gene-specific primers were designed using PrimerBlast software (v2.5.0) and synthesized by Invitrogen (Table 1). Melting curve analysis was performed each time to check the specificity of primers. Each sample was run in triplicate and the relative expression (ΔΔt) of the mRNA was calculated and normalized to the housekeeping gene *GAPDH*.

### 2.4. Statistical Analysis

Unless otherwise stated, data are presented as mean ± standard error of the mean (SEM). Data from four independent experiments were cumulated for the analysis. To determine the significant differences between the groups, normally distributed data were compared using the unpaired Student’s *t*-test. A two-sided *p*-value ≤ 0.05 was considered statistically significant. Analysis was performed using GraphPad Prism (v10).

## 3. Results

**Figure 1:**
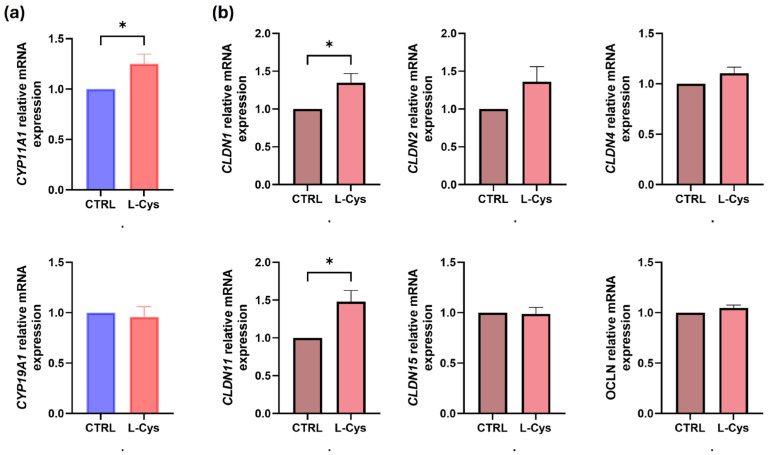
(**a**) Testosterone and (**b**) BTB relative gene expression after human Leydig cells were treated with 500 μM of L-cysteine. * *p* < 0.05.

The human Leydig cells and THP-1 monocytes were treated with L-cysteine for 24 h, followed by measurement of the mRNA expression of testosterone regulatory genes and BTB genes. The L-cysteine treatment did not impact cell viability in either Leydig cells or THP-1 monocytes. L-cysteine (500 µM) significantly upregulated (*p* = 0.03) the mRNA expression of the *CYP11A1* gene in human Leydig cells. Conversely, there was no observed impact on the expression of the *CYP19A1* gene (Figure 1a). Additionally, the expression of the BTB genes *CLDN1* (*p* = 0.03), *CLDN11* (*p* = 0.02) and *TJP1* (*p* = 0.02) was upregulated, while no significant changes were noted in the expression of *CLDN2*, *CLDN4*, and *CLDN15* genes (Figure 1b).

**Figure 2:**
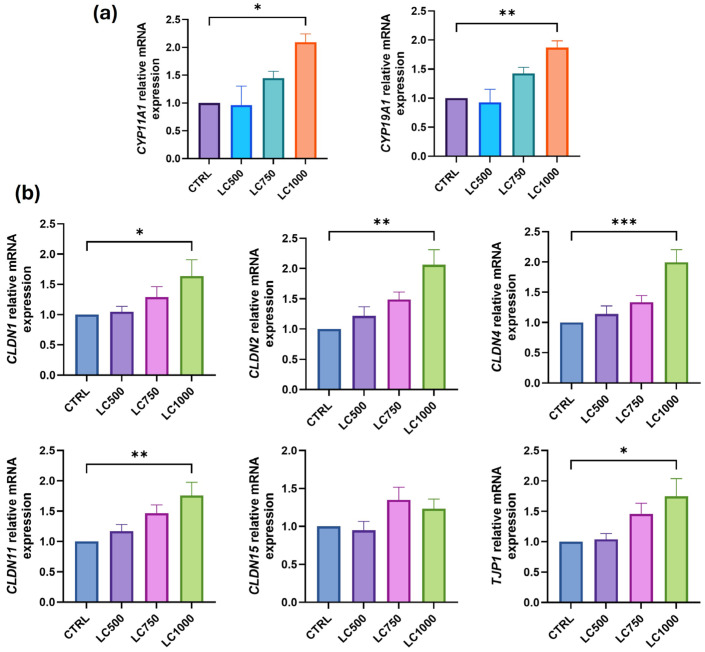
(**a**) Testosterone and (**b**) BTB relative gene expression after THP-1 monocytes were treated with different concentrations of L-cysteine (500–1000 μM). * *p* < 0.05; ** *p* < 0.01; *** *p* < 0.001.

THP-1 monocytes were exposed to varying concentrations of L-cysteine, with the highest concentration (1000 µM) resulting in a significant increase in the expression of *CYP11A1* (*p* = 0.03) and *CYP19A1* (*p* < 0.01) (Figure 2a). Additionally, the expression of BTB genes, *CLDN1* (*p* = 0.04), *CLDN2* (*p* < 0.01), *CLDN4* (*p* < 0.01), *CLDN11* (*p* < 0.01), and *TJP1* (*p* = 0.03) was significantly upregulated after treatment with L-cysteine (1000 µM). However, there was no notable change in the gene expression of *CLDN15* (Figure 2b) and lower concentrations of L-cysteine (500 µM and 750 µM) did not impact the gene expression profile.

**Figure 3:**
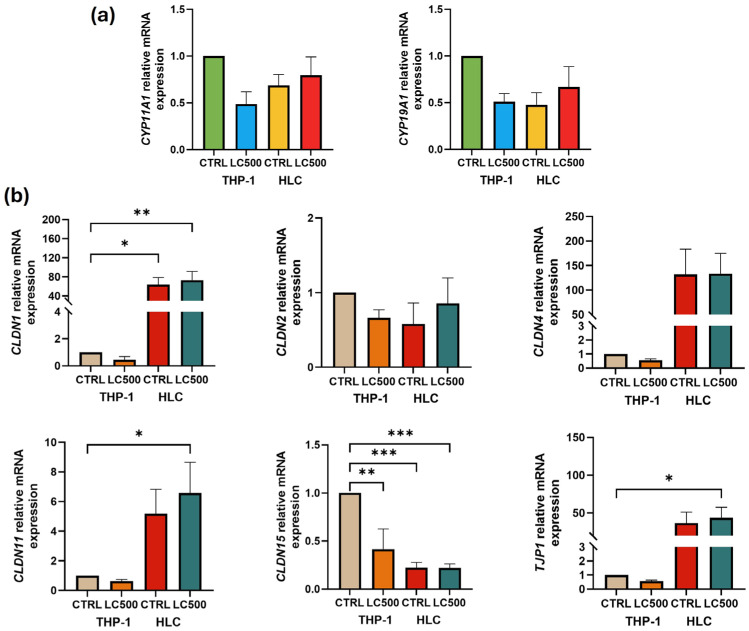
Comparison of (**a**) testosterone regulatory and (**b**) BTB relative gene expression among human Leydig cells and THP-1 after treatment with L-cysteine (500 μM). * *p* < 0.05; ** *p* < 0.01; *** *p* < 0.001.

Gene expression levels were compared between Leydig cells and THP-1 monocytes, revealing higher expression of BTB genes *CLDN1* (*p* < 0.01), *CLDN11* (*p* = 0.03), and *TJP1* (*p* = 0.04) in Leydig cells compared to THP-1 monocytes (Figure 3b). No significant difference was observed in the expression of testosterone-regulatory genes between Leydig cells and THP-1 monocytes (Figure 3a).

**Figure 4:**
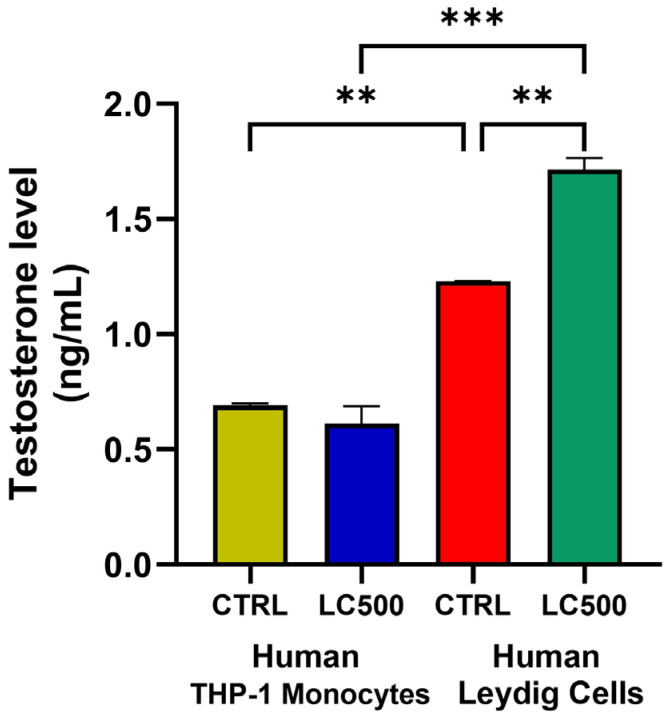
Testosterone concentration in the cell culture supernatant after L-cysteine treatment in human Leydig cells and THP-1 monocytes. ** *p* < 0.01; *** *p* < 0.001.

The testosterone levels in the human Leydig cells and THP-1 supernatants were measured after treatment with L-cysteine. Figure 4 illustrates that L-cysteine (500 µM) significantly increased (*p* < 0.01) the testosterone levels in the Leydig cells. However, L-cysteine treatment did not show any effect on the testosterone secretion in the THP-1 monocytes. The untreated Leydig cells comparatively (*p* < 0.01) secreted more testosterone (2-fold) than the untreated as well as treated THP-1 monocytes.

## 4. Discussion

Approximately 5 million men in the United States, including 20–50% of men aged 60 and older, experience a significant decrease in their serum testosterone levels [26]. Among couples seeking medical assistance for infertility issues, around 15% of men are affected [27]. Hypogonadism, characterized by low testosterone levels, can affect men of various ages. However, primary testicular deficiency, also known as primary hypogonadism, typically does not lead to a change in serum LH levels. The stimulation of testosterone production in response to LH administration is generally lower in older men than in young men, indicating a decline in the responsiveness of Leydig cells with age [28]. LH administration is often unsuccessful in boosting testosterone levels in men due to its inability to stimulate Leydig cell testosterone production. On the other hand, the administration of exogenous testosterone replacement therapy has proven effective in alleviating symptoms associated with low testosterone; nevertheless, it is important to note that men who undergo this therapy face an elevated risk of stroke, heart attack, and prostate cancer [29].

The present study is the first to investigate the effect of L-cysteine treatment on the relative mRNA expression of the testosterone regulatory genes and testosterone secretion in human Leydig cells and THP-1 monocytes. Leydig cells are specialized endocrine cells essential for male fertility due to their involvement in testosterone production and support of spermatogenesis [19]. These cells are susceptible to oxidative stress from reactive oxygen species (ROS) produced by cytochrome 450 enzymes and have a significant detrimental effect on Leydig cell functions on the steroidogenic pathway. Further, due to their proximity to testicular macrophages, potentially leading to damage and dysfunction, they ultimately play a role in male infertility [30]. Furthermore, Leydig cells play a crucial role in the local immune response by releasing cytokines and chemokines, aiding in the defense against pathogens, and maintaining testicular health [31].

Research indicates that there is an increase in superoxide levels, while the antioxidant defense molecules such as glutathione peroxidase and GSH exhibit a decline in aged Leydig cells [32,33]. Furthermore, various studies have documented age-related declines in GSH levels across multiple biological systems, including human serum and the livers and brains of rats [34,35,36]. A decrease in GSH levels within Leydig cells could lead to a decline in steroid hormone production [15].

L-cysteine is a powerful antioxidant and a potential treatment option to reduce the risk of several disease conditions [37,38,39,40]. L-cysteine supplementation helps to restore glutathione synthesis, safeguards the body against diseases caused by imbalanced redox reactions, and counteracts oxidative stress by activating genes [41,42,43]. L-cysteine is widely used in the development of numerous drugs. However, the number of clinical trials testing the effects of L-cysteine on human health and wellness is small. N-acetyl-L-cysteine (NAC) is commonly used to combat inflammation and oxidative stress both in vivo and in vitro [44]. It has the potential to enhance animal reproduction and reproductive performance as a nutritional supplement by improving placental function and regulating hormone production [45]. Furthermore, NAC provides significant protective effects against busulfan-induced male reproductive impairment, possibly through modification of the Nrf2/HO-1 signaling pathway [46]. Co-supplementation with alpha-lipoic acid and NAC prevented intensive swimming-induced testicular spermatogenic and steroidogenic disorders induced by ROS generation [47].

Our study reports that L-cysteine upregulates the steroidogenic gene *CYP11A1* in human Leydig cells and THP-1 monocytes. The overexpression of the *CYP11A1* gene resulted in increased testosterone secretion by L-cysteine-treated Leydig cells in comparison to the untreated cells. *CYP11A1* plays a vital role in converting cholesterol into other intermediates, which subsequently serve as precursors for the synthesis of steroid hormones [6,48]. Previous studies have documented the decline in levels of *CYP11A1* gene expression and testosterone production in aging Leydig cells [49,50]. Despite the decline in steroidogenic enzyme levels in aged cells, the availability of sufficient cholesterol to the inner mitochondrial membrane steroidogenic enzyme *CYP11A1* can still result in high testosterone levels [51]. In addition to sexual function, testosterone increases skeletal muscle mass and strength as well as bone mineral density [52]. The supplementation of L-cystine may promote the expression of the *CYP11A1* gene, potentially leading to an increase in testosterone synthesis. *CYP19A1* catalyzes the final stages of estrogen production and has been identified in the expression of mammalian trophoblasts [53]. Previous studies in male rats supplemented with NAC showed no changes in the expression of the testicular *CYP11A1* gene [38]. However, when NAC was administered to rat granulosa cells, it led to an increase in the gene expression of both *CYP11A1* and *CYP19A1* [54]. A low dose of NAC (1 µM) in porcine placental trophoblast cells upregulated the CYP19A1 gene expression, whereas a high dose of NAC (10 mM) downregulated *CYP11A1* mRNA [45]. In this study, treatment with 500 µM and 1000 µM L-cysteine significantly upregulated the expression of the *CYP11A1* gene in human Leydig cells and THP-1 cells, respectively.

L-cysteine supplementation resulted in an increase in BTB gene expression in both Leydig cells and THP-1 monocytes. Unexplained male infertility can often be linked to endocrine disturbances in testicular development during the neonatal period, which can be influenced by a combination of environmental pollutants, genetic factors, and epigenetic changes. These factors are linked to the BTB, as they are the main target of different environmental toxins [55]. Endocrine-disrupting compounds are believed to cause testicular damage and affect the integrity of the BTB and the endocrine function of Leydig cells [56,57]. The permeability of the BTB is increased by the presence of nanoparticles and ionizing radiation, which induce structural damage. Consequently, this leads to impaired reproductive cell function and a decline in sperm quality [58,59,60]. In addition to maintaining structural integrity, cell junction proteins facilitate several events in spermatogenesis. The function of the BTB in protecting germ cells is compromised with age due to a decrease in BTB proteins, leading to an inability to block the passage of harmful substances into the seminiferous tubules [61]. NAC attenuates the damage caused to the BTB by SR X-rays [62] and may be used as a preventive measure against iron overload-induced testicular damage [41].

The BTB is a complex structure present in the seminiferous tubules of the mammalian testis [62]. It differs from other tissue barriers in that it is composed of four different cell junctions. The BTB is created by tight junctions (TJs), ectoplasmic specializations, desmosomes, and gap junctions (GJs) [63]. Testosterone secreted by Leydig cells in the presence of LH is required for the maintenance of the BTB, spermatogenesis, and fertility. It also promotes both Sertoli-germ cell junction assembly and disassembly [63]. TJ, which has gate and fence functions, is the most important component of the BTB. The gate function prevents the passage of water, solutes, and other large molecules in the paracellular space (by creating a barrier), whereas the fence function restricts the movement of proteins and lipids between apical and basolateral domains (by generating cell polarity) [63]. Factors such as cytokines and tissue damage affect junctional integrity and could enhance the permeability of endothelial and epithelial barriers [64,65,66]. TJs contain many proteins including claudins [67].

We compared the expression levels of these genes in human Leydig cells and THP-1 cells. Interestingly, the *CLDN1*, *CLDN11*, and *TJP1* genes showed significantly higher expression levels in HLC than in THP-1 monocytes. This suggests a potential role for these genes in the specific functions and characteristics of Leydig cells, highlighting the importance of further investigation into their regulatory mechanisms and implications in cellular function. The expansion capacity of human Leydig cells is limited, which means that they cannot be grown for a long duration, so we used THP-1 monocytes as an additional model to study the effect of L-cysteine in the regulation of testosterone synthesis. Due to their homogenous genetic background, the THP-1 monocytes facilitate the reproducibility of the findings by minimizing the effect of variability in the cell phenotype [68]. The concentration of L-cysteine used was similar to those found in the bloodstream [69].

The strength of our study is the novel finding that L-cysteine supplementation significantly upregulated the expression of *CLDN1*, *CLDN11*, and *TJP1* genes in the human Leydig cells, while *CLDN1*, *CLDN2*, *CLDN4*, *CLDN11*, and *TJP1* genes were significantly upregulated in the THP-1 monocytes. Two different cell lines were used to test the hypothesis and compare the expression profile between them. L-cysteine possesses antioxidant properties that can counteract reactive particles, preventing potential damage to cells and tissues [70]. These results indicate that L-cysteine has the potential to serve as a beneficial supplement for controlling the expression of BTB genes, which can mitigate the harmful effects of oxidative stress and hinder the entry of cytotoxic drugs into the seminiferous tubules through the BTB. This has the potential to improve spermatogenesis and the development of germ cells. Clinical studies are required to determine whether L-cysteine co-supplementation can benefit aging men by increasing the integrity of the BTB and boosting testosterone production. If so, co-supplementation with L-cysteine could provide a safer alternative to treatment with testosterone alone, which suppresses LH levels. Being able to explore the development of new therapies to increase intratesticular bioactive androgen levels without any side effects can help reduce infertility. The strength of our study is that we have used Human Leydig cells; however, these cells present significant challenges in terms of growth and culture. Their slow proliferation and restricted expansion capacity resulted in a limited number of cells available for experimentation, leading us to employ a single concentration of L-cysteine (500 µM) for treatment, with untreated Leydig cells serving as a control. Different concentrations of L-cysteine could have provided more comprehensive data. In contrast, THP-1 cells exhibited a more rapid growth rate, allowing for the examination of multiple L-cysteine concentrations. Additionally, a limitation of this study is the absence of concurrent analysis of protein levels in the treated cells.

## 5. Conclusions

**Figure 5:**
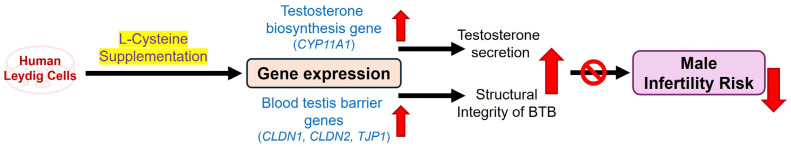
Summary diagram illustrating that L-cysteine supplementation increased the gene expression of testosterone biosynthesis and BTB genes and enhanced testosterone secretion.

Leydig cells, also known as the interstitial cells of the testes, are found adjacent to the seminiferous tubules in the testicle and are vital for testosterone biosynthesis. Previous studies have documented an association between the decline in levels of *CYP11A1* gene expression and testosterone production in aging Leydig cells and infertility. Disruption of any of these interactions can lead to impaired spermatogenesis and ultimately affect male fertility. This study demonstrates that the supplementation of L-cysteine leads to augmented expression of the genes associated with testosterone and BTB in both human Leydig cells and THP-1 monocytes. Supplementation with L-cysteine resulted in upregulation of the testosterone biosynthesis gene and testosterone secretion, which can potentially enhance spermatogenesis (Figure 5). Clinical studies are necessary to investigate the potential advantages of L-cysteine co-supplementation for elderly males, focusing on strengthening the integrity of the blood–testis barrier and elevating testosterone levels to reduce the likelihood of male infertility.

## Figures and Tables

**Table 1:** List of primers used for qPCR analysis.

Gene	Orientation	Primer Sequence (5′–3′)
*CYP11A1*	Forward	CGTCAGATCCATCGGGTTAATG
	Reverse	CATTCCAACCATCCAGGTATCG
*CYP19A1*	Forward	GAGAACCAGGCTACAAGAGAAA
	Reverse	TGGTGGAATCGGGTCTTTATG
*CLDN1*	Forward	CCAGTTAGAAGAGGTAGTGTGAAT
	Reverse	CAGCCAGCTGAGCAAATAAAG
*CLDN2*	Forward	GGTGACATCCAGTGCAATCT
	Reverse	CTACCGCCACTCTGTCTTTG
*CLDN4*	Forward	CAACTGCCTGGAGGATGAAA
	Reverse	CACCGGCACTATCACCATAAG
*CLDN11*	Forward	GTGTGATCTCGGCTCATGTA
	Reverse	GTAGTAGTGAACGCCTGTAGTC
*CLDN15*	Forward	TGACTCTGCCAAACAGCTAC
	Reverse	GTCGGTGGCACAGCTAAA
*TJP1*	Forward	CCTTAGTGTCCAAACCAGACC
	Reverse	AAAGCTGCCTGAGCAGTATC
*OCLN*	Forward	CATTGCCATCTTTGCCTGTG
	Reverse	CCAAAGCCACTTCCTCCATAA
*GAPDH*	Forward	AGTATGACAACAGCCTCAAGAT
	Reverse	GTCCTTCCACGATACCAAA

## Data Availability

The original contributions presented in the study are included in the article; further inquiries can be directed to the corresponding author.
